# Measuring RNA UNCG Tetraloop Refolding Dynamics Using Temperature-Jump/Drop Infrared Spectroscopy

**DOI:** 10.1021/acs.jpclett.2c02338

**Published:** 2022-09-27

**Authors:** C. P. Howe, G. M. Greetham, B. Procacci, A. W. Parker, N. T. Hunt

**Affiliations:** † Department of Chemistry and York Biomedical Research Institute, University of York, Heslington, York YO10 5DD, U.K.; ‡ Central Laser Facility, Research Complex at Harwell, STFC Rutherford Appleton Laboratory, Harwell Oxford, Didcot, Oxon OX11 0QX, U.K.

## Abstract

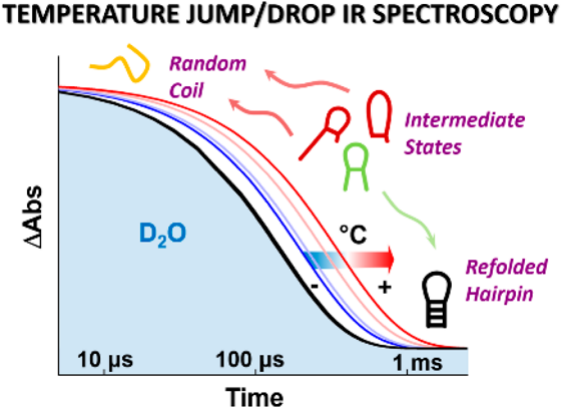

Determining the structural dynamics of RNA and DNA is essential to understanding their cellular function, but direct measurement of strand association or folding remains experimentally challenging. Here we illustrate a temperature-jump/drop method able to reveal refolding dynamics. Time-resolved temperature-jump/drop infrared spectroscopy is used to measure the melting and refolding dynamics of a 12-nucleotide RNA sequence comprising a UACG tetraloop and a four-base-pair double-stranded GC stem, comparing them to an equivalent DNA (TACG) sequence. Stem-loop melting occurred an order of magnitude more slowly in RNA than DNA (6.0 ± 0.1 μs versus 0.8 ± 0.1 μs at 70 °C). In contrast, the refolding dynamics of both sequences occurred on similar time scales (200 μs). While the melting and refolding dynamics of RNA and DNA hairpins both followed Arrhenius temperature dependences, refolding was characterized by an apparent negative activation energy, consistent with a mechanism involving multiple misfolded intermediates prior to zipping of the stem base pairs.

Ribonucleic acid (RNA) sequences and their deoxy counterparts (DNA) are fundamental to the storage, regulation, and expression of genetic information in organisms. Revealing the underlying molecular mechanisms is central to our understanding of biological processes as well as supporting development of new chemical and biomedical technologies, such as nucleic acid aptamers and antisense therapeutics.

While the structures of nucleic acid sequences are well understood, the dynamic fluctuations and interactions within and between nucleic acid strands in solution remain a source of uncertainty. Insights have been gained by nonequilibrium temperature jump (T-jump) spectroscopy experiments, which induce a rapid rise in temperature as a means to initiate and follow conformational changes in real time.^1−10^ T-jump methods have been used to investigate the impact of a number factors upon nucleic acid dynamics, including base sequence, chain length, and the presence of small molecule ligands.^1−3,5,6^ Typically, T-jump studies employ fast (nanosecond) initiation, but the samples cool on multi-millisecond time scales. The latter are too slow to observe refolding directly, meaning that refolding (association) rates have to be inferred from two state models linking the dissociation rate to the equilibrium constant.^5,7^ Here, we demonstrate an approach that exploits a much faster sample cooling time to enable observation of both the melting and refolding dynamics of nucleic acid tetraloop hairpins following T-jump initiation.

To date, much emphasis has been placed upon elucidating the dynamics of double-stranded DNA,^1−6,10^ but RNA demonstrates greater complexity in the biological setting due to its propensity to exist as single strands that fold into functional three-dimensional structures. Hairpins, consisting of a Watson–Crick base paired stem and a single-stranded loop, are among the most common RNA structural motifs, occurring in a range of prokaryotic and eukaryotic RNAs.^11^ Tetraloops in particular provide nucleation sites for folding and recognition sites for RNA binding proteins.^12,13^ The two most common loop motifs are GNRA and UNCG, where R = purine and N = any nucleotide.^12^ The latter are more thermodynamically stable, displaying melting points some 20 °C higher than other sequences, whereas the former are more prevalent, participating in tertiary interactions and protein recognition.^12^

The melting characteristics of UNCG loops have been determined previously by static spectroscopy experiments,^14,15^ but the folding dynamics have yet to be resolved. To address this important issue, we combine a nanosecond T-jump, to initiate melting, with a subsequent sample cooling rate that is fast enough to allow observation of strand refolding. Faster cooling is achieved using a thin cell to enhance heat transfer. Using IR spectroscopy as a probe of vibrational modes of the nucleic acid bases provides real-time insight into stem base pairing and stacking. Although DNA tetraloops are not known biologically, comparing RNA tetraloop dynamics with those of an analogous DNA sequence reveals inherent differences between molecules with overtly similar molecular structures.

The salt-free, lyophilized DNA and RNA oligomer sequences 5′-GCGC(XACG)GCGC-3′ (RNA: X = U; DNA: X = T; “(...)” indicates the loop position) were purchased from Eurogentec. Other chemicals were purchased from Sigma-Aldrich and used without modification. The GCGC stem sequence of the tetraloops was chosen to minimize base pair slippage. The reduction in the molar extinction coefficient of the guanine ring vibrational mode (G_R_) observed at 1575 cm^–1^ upon base stacking provided a spectral marker for stem melting and reassociation.^1−4,16−20^

All DNA and RNA samples were prepared to a strand concentration of 10 mM, which is below the threshold for duplex formation, in 1 M deuterated phosphate buffer (pD 6.8).^15^ For all spectroscopy measurements a 15–20 μL aliquot was placed in a temperature-controlled cell (Harrick, ±1 °C) equipped with CaF_2_ windows. A 50 μm PTFE spacer defined the path length for IR absorption measurements while a 12 μm path length was used for T-jump experiments.

IR absorption spectra were measured using a Bruker Vertex 70 Fourier transform (FT)-IR spectrometer. The T-jump measurements were performed using the STFC Central Laser Facility’s ULTRA spectrometer.^1,21,22^ Briefly, a 4 ns duration T-jump pump pulse (125 Hz), generated by a Nd:YAG-pumped OPO, centered at 2750 cm^–1^, resonant with the high-frequency wing of the OD-stretching vibration of the solvent initiated a T-jump of 10 °C in the sample (averaged across the sample), as determined by trifluoroacetic acid (TFA) calibration.^1,21,22^ A series of probe pulses centered at 1650 cm^–1^ (bandwidth: 300 cm^–1^) generated by a Ti:sapphire-pumped OPA (10 kHz) with difference frequency mixing of signal and idler were used to capture the nanosecond to millisecond nucleic acid dynamics using the time-resolved multiple probe (TRMPS) method.^1,21,22^ Data were collected at T-jump-probe time delays from 1 ns to 8 ms, though the cooling of the sample was complete by 4 ms (vide infra) such that data obtained at time delays from 4 to 8 ms were used for background correction.^1^

Infrared absorption spectroscopy showed that increasing the temperature of the RNA hairpin sample from 20 to 80 °C under equilibrium (non T-jump) conditions induced changes in band position and intensity across the 1550–1700 cm^–1^ region that are broadly similar to those observed for DNA samples (Figures 1a,b and S1). The temperature-induced spectral changes are represented as difference spectra relative to the 20 °C measurement. By comparison with previous work the changes in this spectral region can be assigned to the melting of double-stranded GC-rich nucleic acids.^16^ We will focus on the guanine ring vibrational mode (G_R_) at 1575 cm^–1^ (purple panels in Figures 1 and S1). This band increases markedly in intensity with temperature, and we assign this to the loss of base stacking and pairing in the stem of the loop.^16^ By extension, we assume that loss of the stem structure leads to melting of the hairpin loop.

**Figure 1:**
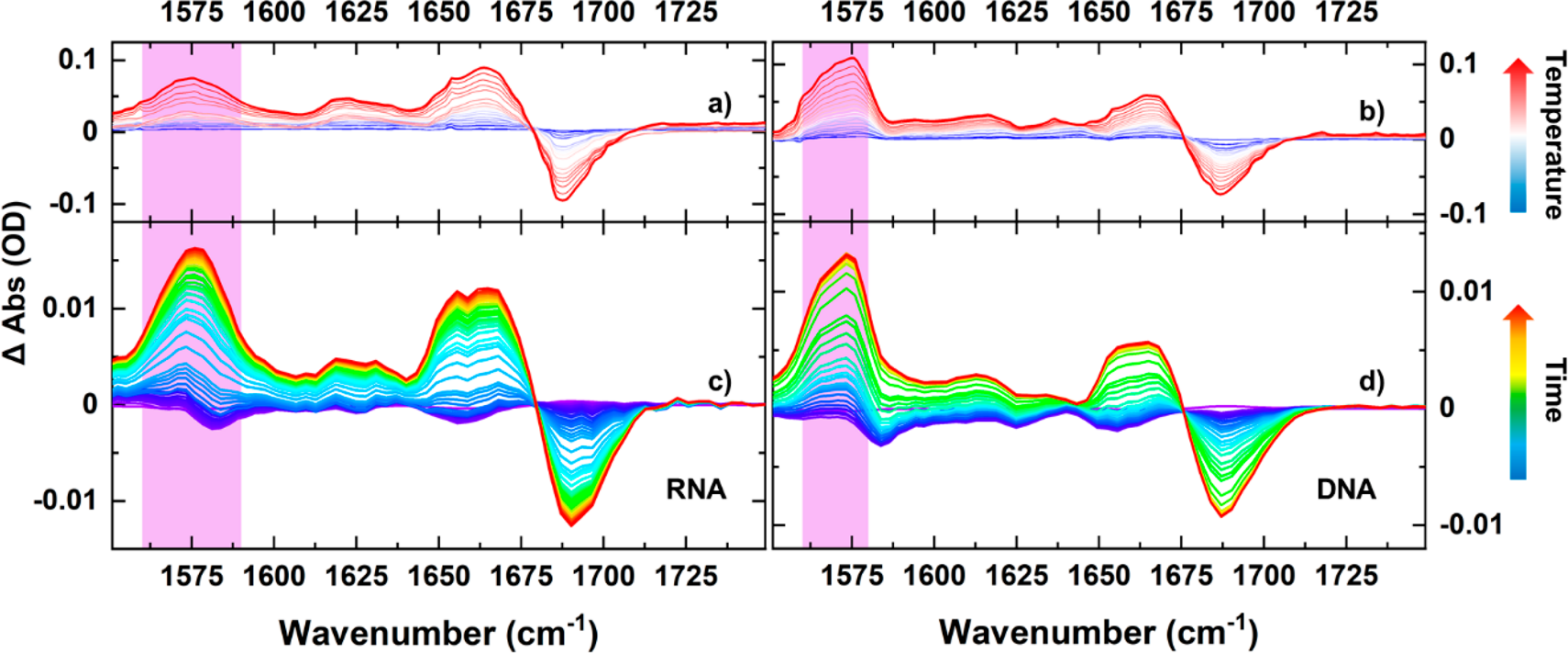
IR absorption (a, b) and T-jump IR spectra (c, d) of RNA (a, c) and DNA (b, d) tetraloop hairpins. (a) and (b) show solvent-corrected FT-IR difference spectra as a function of *T* relative to the spectrum at 20 °C of (a) the RNA and (b) DNA tetraloops. The color scale runs from 20 to 80 °C (blue–red) with the G_R_ mode highlighted. Panels c and d show the T-jump IR spectra of RNA and DNA, respectively (*T*_0_ = *T*_m_ – 5 °C). Time delays from 1 ns (blue) to peak signals (red) at 20 μs (RNA) and 6 μs (DNA) are displayed. The T-jump data are represented as T-jump on–T-jump off difference spectra; the increase in amplitude of the G_R_ band upon melting of the double-stranded GCGC stem appears as a positive peak highlighted by the purple panel. The negative spectral features at very early time in the T-jump data are due to a fast hydrogen-bonding rearrangement. For visual clarity only, T-jump spectra have been baseline corrected to account for solvent-related effects.

Melting curves for the RNA and DNA hairpins were obtained by plotting the absorbance of the G_R_ mode as a function of temperature (Figure S2). A Van’t Hoff analysis was used to obtain thermodynamic parameters associated with the melting transition (Table 1). Because these systems are monomolecular and below the threshold concentration for duplex formation, the melting temperature of the sequences is concentration-independent.^15^

**Table 1:** Thermodynamic Parameters Obtained via Van’t Hoff, Arrhenius, and Eyring Analyses

		RNA	DNA	
	*T*_m_	81 °C	76 °C	
Van’t Hoff	Δ*H*	118.3	124.2	kJ mol^–1^
	Δ*S*	334	356	J K^–1^ mol^–1^
	Δ*G*^a^	14.8	13.9	kJ mol^–1^
Arrhenius	*E*_a,m_	83.3	64.0	kJ mol^–1^
	*E*_a,r_	–48.2	–45.2	kJ mol^–1^
Eyring	Δ*H*_m_^‡^	80.4	61.2	kJ mol^–1^
	Δ*S*_m_^‡^	86	46	J K^–1^ mol^–1^
	Δ*G*_m_^‡^^a^	53.8	46.8	kJ mol^–1^
	Δ*H*_r_^‡^	–51.2	–48.1	kJ mol^–1^
	Δ*S*_r_^‡^	–324	–315	J K^–1^ mol^–1^
	Δ*G*_r_^‡^^a^	49.1	49.5	kJ mol^–1^

a All Δ*G* calculated at 37 °C.

The results of T-jump spectroscopy measurements on RNA and DNA hairpins are shown in Figure 1c,d. The data were obtained with an initial sample temperature, prior to the T-jump (*T*_0_), 5 deg below the melting temperature of the sample (*T*_0_ = *T*_m_ – 5 °C). The spectra are represented as T-jump on–T-jump off difference spectra and show changes in band positions and intensities that emerge over a period of up to 20 μs following the T-jump (blue–red). For both RNA and DNA samples, the spectral changes following the T-jump were observed to be identical with those in the difference IR absorption spectra (Figure 1a,b) and therefore are assigned to melting of the stem of the loop as the hairpin responds to the change in sample temperature. Comparing the maximum intensity change of the G_r_ band in the T-jump experiment to the change in the same band obtained via FT-IR spectroscopy over the same temperature range showed that the extent of melting achieved for RNA and DNA hairpins was 83% and 98%, respectively.^2^ This establishes that the hairpin samples undergo virtually all of the melting expected over the temperature range during the T-jump experiment. On longer time scales the signals decayed to the baseline as the sample cooled following the T-jump. (Figure S3)

To examine the dynamics of the hairpins following the T-jump, experiments were conducted on the RNA and DNA sequences starting from a range of different initial temperatures (*T*_0_). In each case, the G_R_ band intensity was observed to rise, peaking near 20 and 6 μs for RNA and DNA hairpins, respectively, before decaying on millisecond time scales (Figures 2 and S4). In each case, the temporal dependence of the G_R_ band was fitted using a triple-exponential function (Table S1). The majority of the temporal profile was accounted for by two processes with lifetimes quantifying the rise (τ_1_) and decay (τ_2_) of the signal. The third exponential term was generally of low amplitude (<25%) but was essential to achieve a good quality fit. The triple-exponential method was found to provide more robust results than fitting the data with a stretched exponential function. The results from the fitting process are shown in Figure 3. It is noted that D_2_O gives rise to a small, broadband solvent-dependent contribution to the data. In the analysis that follows this has not been subtracted, but comparisons showed that applying this correction led to changes in lifetime parameters that fell within the errors stated.

**Figure 2:**
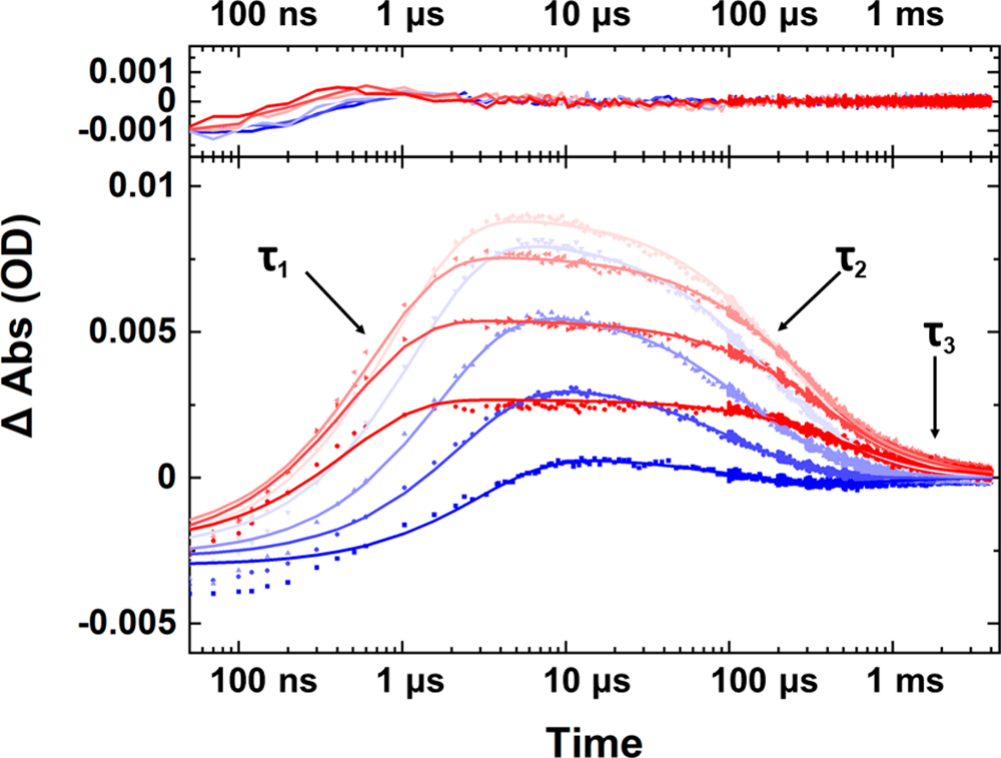
Exemplar data showing the temperature and time dependence of the G_R_ band intensity (dots, bottom) for the DNA hairpin. Data are shown from a *T*_0_ of 50 to 85 °C (blue–red) at intervals of 5 °C through the hairpin melting transition, along with the triple-exponential fits (lines, bottom) and residuals (top). The lifetimes are indicated for the rise (τ_1_) and decay (τ_2_). The third lifetime is a low-amplitude contribution.

**Figure 3:**
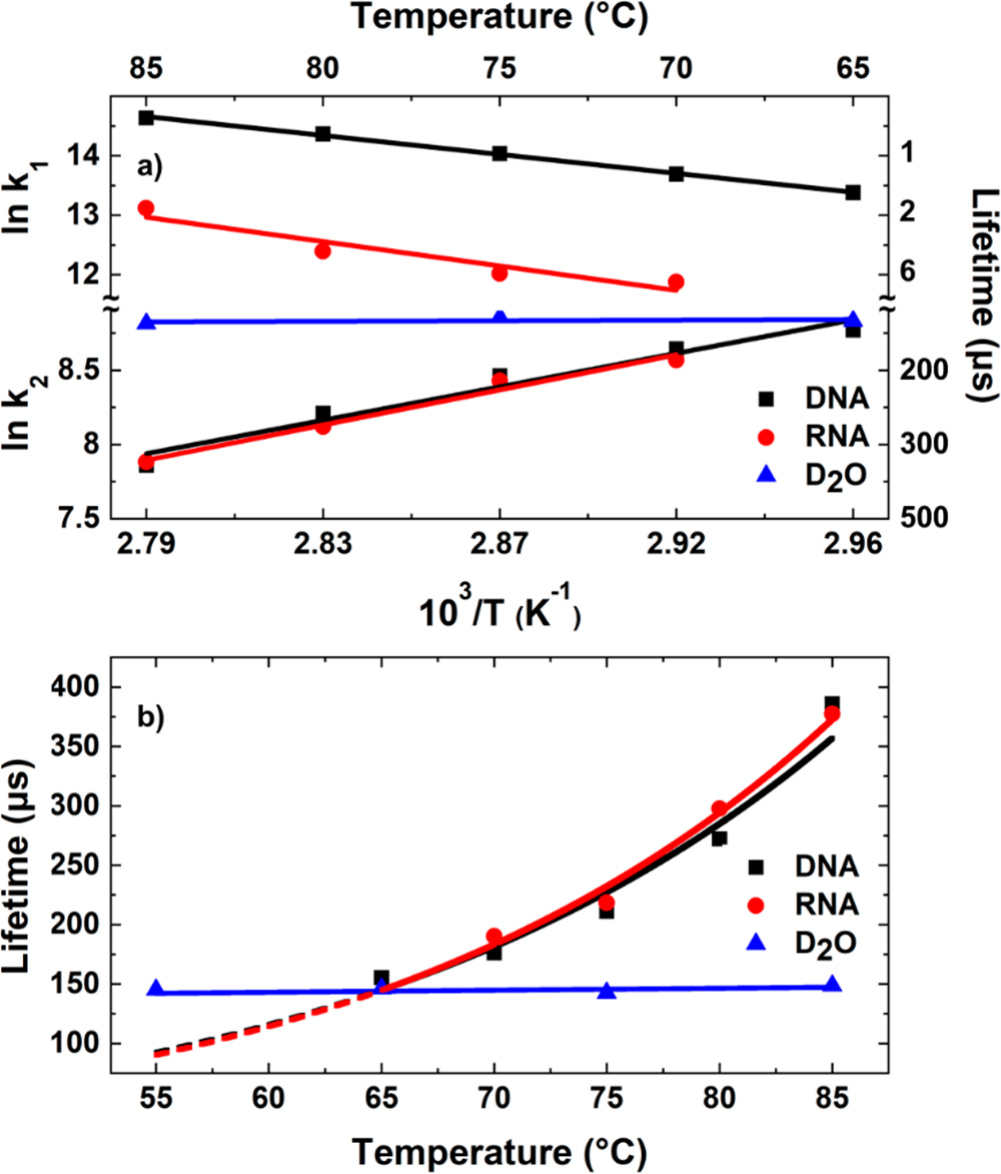
Results of fitting T-jump spectroscopy obtained at a range of *T*_0_ values for RNA and DNA samples. The data are represented using Arrhenius plots showing the temperature dependence of the cooling rate of D_2_O (blue) alongside the melting (a, above D_2_O) and refolding (a, below D_2_O) of DNA (black) and RNA (red) hairpins. In each case, the temperature plotted is *T*_0_ + 5 °C, the average temperature over the T-jump. To focus on experiments where a significant proportion of hairpin melting occurred, data are shown over the temperature range where the maximum change in intensity of the G_R_ band was greater than 20% of the largest signal observed (DNA *T*_0_ + 5 °C, 65–85 °C; RNA 70–85 °C). (b) Temperature-dependent lifetimes for the refolding processes (τ_2_) of DNA (black) and RNA (red) are plotted on a linear axis alongside the temperature invariant cooling time scale obtained for D_2_O (blue, with linear fit). Arrhenius fits (lines, black and red) are shown and extrapolated for temperatures below 20% of the maximum melting signal and where expected lifetimes are faster than the experimental lower limit of the D_2_O cooling lifetime (dotted lines).

In accordance with previous work, we associate the time scale of the rising G_R_ signal, τ_1_, with melting of the GCGC stem, which in this case leads to hairpin loop melting. For RNA hairpins, τ_1_ was found to vary from 2.0 to 6.9 ± 0.6 μs over a range of *T*_0_ values from 80 to 65 °C around the melting transition, while for DNA time scales from 0.4 to 1.5 ± 0.2 μs were observed over the same temperature range. The temperature dependence of the melting time scales for both hairpins (τ_1_) was found to be in good agreement with an Arrhenius representation (Figure 3a, above D_2_O), yielding positive activation energies (*E*_a,m_ = 83 ± 20 and 64 ± 10 kJ mol^–1^ for RNA and DNA hairpins, respectively), as would be expected for strand melting. These values are in good agreement with prior work.^5,23^

Examining the response of the hairpins on microsecond to millisecond time scales, as the sample cools following the T-jump, revealed that the temperature dependence of τ_2_ was also well-represented by Arrhenius behavior. In contrast to τ_1_, however, the time scales of τ_2_ were found to increase with increasing *T*_0_, yielding values from 150 to 400 μs, from 60 to 80 °C for both RNA and DNA, consistent with a process with an apparent negative activation energy (Figure 3a, below D_2_O).

For comparison with the RNA and DNA data, the cooling dynamics of the D_2_O solvent were characterized using T-jump spectroscopy of a D_2_O/TFA solution. Fitting the temporal profile of a vibrational mode of the TFA calibrant molecule at 1670 cm^–1^ to a biexponential function (Figures 3 and S5) has been shown to provide a reliable measure of sample temperature.^1^ A biexponential function was used to mirror the approach used for nucleic acid dynamics while accounting for the fact that the rise time of the T-jump in the solvent is essentially instantaneous, following the profile of the nanosecond laser pulse. For the TFA data, the second time scale recovered from the exponential function was equivalent to the small τ_3_ contribution observed for RNA and DNA, and so we use the major TFA relaxation time scale as an indicator of sample cooling time. The results showed that the cooling time scale of the solvent was invariant with *T*_0_ (Figure 3, blue). To confirm that these results were consistent in samples containing hairpins, the cooling dynamics of a band due to D_2_O (1470 cm^–1^) present in both the TFA calibration sample and the hairpin samples were compared and found to display similar dynamics (Figures S4 and S5).

In addition to showing no Arrhenius behavior, it is clear from Figure 3 that the cooling of the solvent occurs on time scales significantly faster than the decay of the oligonucleotide signals. While D_2_O cooling takes place on <150 μs time scales, the hairpin dynamics of both RNA and DNA show dynamics on several hundreds of microsecond time scales, increasing exponentially as *T*_0_ increases (Figure 3b), which we show below is consistent with a process exhibiting an apparent negative activation energy. As the value of *T*_0_ decreases, the time scales for RNA and DNA signal recovery decrease such that at a *T*_0_ of 65 °C the hairpin dynamics match those of the solvent. We thus conclude that at *T*_0_ values equal to or greater than 70 °C the experiment is probing the native dynamics of the hairpin, which are independent of the solvent behavior. Specifically, this implies that hairpin refolding dynamics are being observed. It is important to note that the spectral changes taking place during the cooling are the reverse of those observed during melting, supporting this assignment (Figure S3).

Using an Arrhenius analysis produced values of −48.2 ± 5.0 and −45.2 ± 5.6 kJ mol^–1^ for the activation energies of refolding (*E*_a,r_) of RNA and DNA, respectively. These values are consistent with negative activation energies that have been calculated for the refolding of oligonucleotides in other studies, confirming that our approach is directly measuring hairpin refolding as well as the validity of the models used to obtain refolding time scales.^3−5,7,23−27^ It is noteworthy that although RNA and DNA show very different melting behaviors, the refolding process is consistent between the two—an outcome which has been reported by previous studies on different sequences and suggests the key rate-limiting steps are conserved in both.^28^ It is important to note that the refolding process as measured is a convolution of the solvent cooling and the nucleic acid dynamics. When these time scales are well separated, as at high temperatures here, the results will be dominated by the nucleic acid dynamics. At lower temperatures a degree of convolution may affect the absolute quantitative accuracy, but even under these conditions, a direct experimental measurement obtained for different molecules under identical experimental conditions will yield valuable comparison data.

The small time scale component (τ_3_) was found to be on the order of 800 μs. On the basis of its magnitude, in nucleic acid samples, which was greater than the solvent signal, and its nonconstant temperature dependence, this contribution could not be attributed simply to solvent response or a cooling property of the cell. As such it may indicate a slower dynamic process that follows the initial refolding, but the small nature of the contribution precludes clear conclusions.

The marked differences in the melting time scales of the RNA and DNA hairpins, 6.0 ± 0.1 vs 0.8 ± 0.1 μs at a *T*_0_ of 70 °C, respectively, indicate that specific structural factors must influence the energetic barrier of the rate-limiting step in the breaking down of the base-paired stem. While the differences between RNA and DNA at the molecular level are limited to a 2′-OH group on the ribose moiety, it is perhaps relevant that they typically adopt different helical geometries in solution. Specifically, the packing of the phosphate groups in the backbone and the stacking of the nucleotide bases are tighter in RNA than in DNA.^29^ X-ray structures of RNA have also determined that individual water molecules link the 2′-hydroxyl groups of adjacent nucleotides and in general have more extensive, ordered hydration of the backbone.^30^ In solution, this has been shown to correlate with additional coupling between the vibrational modes of the backbone in RNA compared to DNA.^31,32^ Such changes in hydration could have implications for the overall stability of the hairpin structure and its dynamics. In particular, the melting process is a combination of the breakdown of base-pairing and base-stacking, but the association and dissociation of the base stacks have been suggested to be slower than base-pairing.^33−35^ This implies that the conformation and hydration dynamics that provide added stability for RNA also influence the relative stability of the base-stacking compared to DNA. The structure of some UNCG tetraloops is also known to specifically slow melting, and this may have a greater effect in RNA.^36^

In contrast to melting, the refolding processes for the RNA and DNA hairpins occur on the same time scales. This observation of an apparent negative activation energy, in accordance with various studies, shows that this follows a more complex mechanism than melting.^3−5,24,33^ The appearance of a rate that decreases with increased temperature has been associated with a rugged potential energy landscape such that refolding is not a simple two-state transition.^7,9,37,38^ Although there are differences between diffusive reassociation of double-stranded DNA and refolding of a hairpin loop, some common elements are to be expected. To refold from a random coil, the hairpin must first make an initial base-pairing contact. There are a number of ways in which a base pair may be mismatched in such a way that it is in fact not possible for the stem sequence to fully base pair, and therefore these are likely to return to the random coil conformation on a number of occasions before full refolding occurs.^7^ Additionally, the initial base pair must be stabilized by base stacking to allow the full zipping of the stem.^33^ Because the rate of base-stacking association is relatively slow, this is likely to result in even correct initial base pairing contacts more often returning to random coil than actually resulting in the full refolding of the stem loop. With many potential states on the energy landscape, the zipping of the hairpin represents a significant loss of entropy, which, despite also leading to a lower enthalpy, results in a free energy barrier.^5,7,24^ A consequence of these competing rates is that when the temperature is increased, the rates of dissociation of base pairs and stacks are increased, making the conjunction of the two less likely and resulting in an overall slower refolding rate.^33,39^ This can be confirmed by applying an Eyring rather than Arrhenius analysis, which gives access to activation enthalpy and entropy values. For the refolding process a negative enthalpy change of activation is observed that is identical with the Arrhenius activation energy (−51.2 ± 5.0 and −48.1 ± 5.6 kJ mol^–1^ for RNA and DNA, respectively). This is accompanied by a negative activation entropy (−324 ± 37 and −315 ± 43 J K^–1^ mol^–1^), such that the Gibbs free energy of activation is positive at elevated *T*, consistent with the observations. The fact that this negative activation energy and indeed the time scale of refolding are shared between both the RNA and DNA hairpin suggests that the rate-limiting step for both is the same—the re-forming of the base stacks. Furthermore, this slow rate of formation of the base stacks has a more significant impact on the refolding process than the differing features of RNA and DNA.

In conclusion, we have demonstrated that it is possible to apply T-jump time-resolved spectroscopy strategies to observe the refolding dynamics of nucleic acid hairpins. Using a system in which the solvent cooling time is shorter than the refolding time of the sample effectively produces a combined T-jump followed by a T-drop experiment, making it possible to measure the native dynamics of both RNA and DNA hairpin melting and refolding. This provides a direct measurement of parameters that previously had to be calculated from the melting rates and equilibrium constants. The use of IR spectroscopy for label-free detection of the conformational changes will also enable future comparisons with, for example, refolding rates obtained via microfluidic temperature drop techniques employing incorporation of fluorescent labels.^40^ We have shown previously that the cooling time scale for a sample varies sensitively with the sample path length, meaning that this strategy can be used to tune the cooling time to a certain degree to match the dynamics of the analyte molecule.^21^

Comparing our results obtained on similar RNA and DNA hairpins reveals distinct melting dynamics, with RNA melting an order of magnitude more slowly than DNA, which we attribute to the influence of the helical conformation on the stabilization of the base stacking. However, the refolding dynamics both occur on similar time scales and show behavior which is consistent with a negative activation energy. These observations are in good agreement with previous studies and give insights into the energy landscape that result in the similar refolding dynamics of the DNA and RNA hairpins.

## Supporting Information Available

Sample preparation details and additional experimental details; additional spectroscopy data: temperature-dependent FT-IR spectra of all samples, T-jump dynamics for nucleic acid hairpins and solvent (PDF)
