# Supplementary material for: Measuring RNA UNCG Tetraloop Refolding Dynamics Using Temperature-Jump/Drop Infrared Spectroscopy
Source: J Phys Chem Lett. 2022 Sep 27;13(39):9171–6. doi: 10.1021/acs.jpclett.2c02338 (PMC9549515; doi:10.1021/acs.jpclett.2c02338)
Supplement: Supplementary file 1 — jz2c02338_si_001.pdf [file jz2c02338_si_001.pdf]

Measuring RNA UNCG Tetraloop Refolding Dynamics Using Temperature-Jump/Drop Infrared Spectroscopy

Supporting Information

C.P. Howe,[1] G.M. Greetham,[2] B. Procacci,[1] A.W. Parker[2] and N.T. Hunt[1]

1) Department of Chemistry and York Biomedical Research Institute, University of York, Heslington, York, YO10 5DD, UK

2) Central Laser Facility, Research Complex at Harwell, STFC Rutherford Appleton Laboratory, Harwell Oxford, Didcot, Oxon, OX11 0QX, UK.

Sample Preparation:

DNA and RNA Oligomers (5'-GCGCTACGGCGC-3' and 5'-GCGCUACGGCGC-3') were purchased as salt free, lyophilised solids from Eurogentec. All other chemicals were purchased from Sigma-Aldrich and used without further purification. Solutions of each oligomer were prepared to 10 mM using a deuterated 1 M Phosphate buffer (pD 6.8). Hairpins were annealed by heating to 95 °C for 5 min and leaving to cool for 1 hour.

IR absorption spectroscopy and Oligomer melting:

IR absorption spectra were measured using a Bruker Vertex 70 FT-IR spectrometer with a resolution of 1 cm^{-1}. Samples were prepared by placing 15-20 µl solution in a temperature controlled (to ±1 °C) Harrick Cell with 2 mm thick CaF_2 IR windows. A 50 µm PTFE spacer was used to define the path length.

T-jump Pump – Probe spectroscopy

The T-jump was conducted using the ULTRA laser system at the Central Laser Facility of the Rutherford Appleton Labs using the T-jump and Time Resolved Multiple Probe (TRMPS) method described previously. [4–7]

T-jump pulses were produced at a repetition rate of 1 kHz using a home-built Nd:YAG-pumped optical parametric oscillator (OPO). Each pulse had a pulse length of 4 ns and an energy of 70 µJ. This was then optically chopped down to 125 Hz, resulting in a measurement of 8 ms, with the final 4 ms used as the pump-off, which significantly reduces the instrumental noise in the data. The T-jump pump was tuned to the high frequency edge of the of the OD stretching vibrational mode (2750 cm^{-1}) so that transmission through the sample was ~30%, with a T-jump of ~10 °C as calibrated by TFA.

Probe pulses were produced by a regeneratively-amplified Ti:S laser pumping a white-light-seeded optical parametric amplifier with difference frequency mixing of the signal and idler outputs. The probe pulses for the T-jump were produced at a pulse repetition rate of 10 kHz (once every 100 µs), with a pulse duration of 50 fs and a centre frequency of 1650 cm^{-1} (bandwidth: 300 cm^{-1}) to capture the changes in the nucleotide base vibrational modes of DNA and RNA. With the addition of an electronic delay to change the relative triggering of the pump and probe pulses, it was possible to take measurements from 1 ns to 4 ms.

A fraction of the probe light was split before the sample and dispersed on a separate detector to generate a reference to subtract from the sample data to remove any instrumental noise and fluctuations.

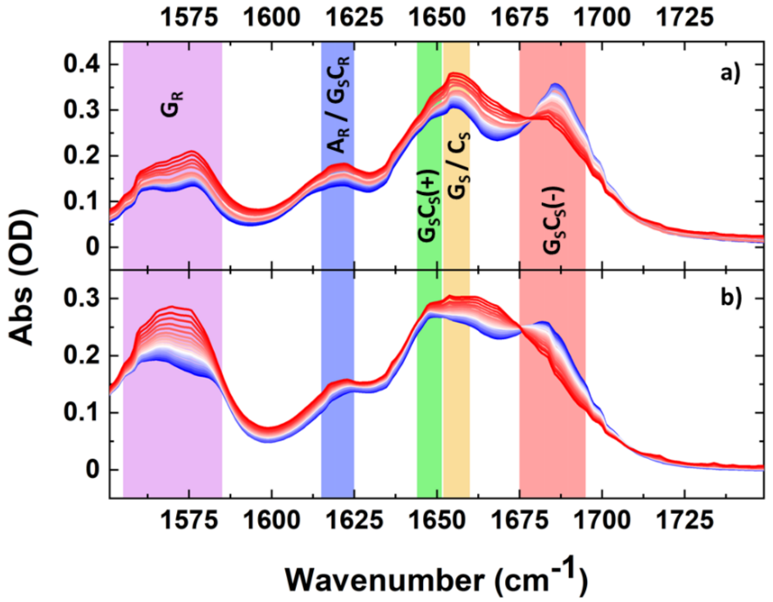

Figure S1. FT-IR spectra of tetraloop samples. Data are shown for a) RNA and b) DNA from 20 to 80 °C (blue-red). Prominent vibrational modes are indicated by colored panels: G_R (1575 cm^{-1}, purple), A_R and G_SC_R (1620 cm^{-1}, blue), G_SC_S(+) (1648 cm^{-1}, green), G_S and C_S (1656 cm^{-1}, yellow) and G_SC_S(-) (1686 cm^{-1}, red).[1–3] The notation used to identify the vibrational modes of the bases is consistent with that used in Refs 2&3. Spectra have been solvent corrected.

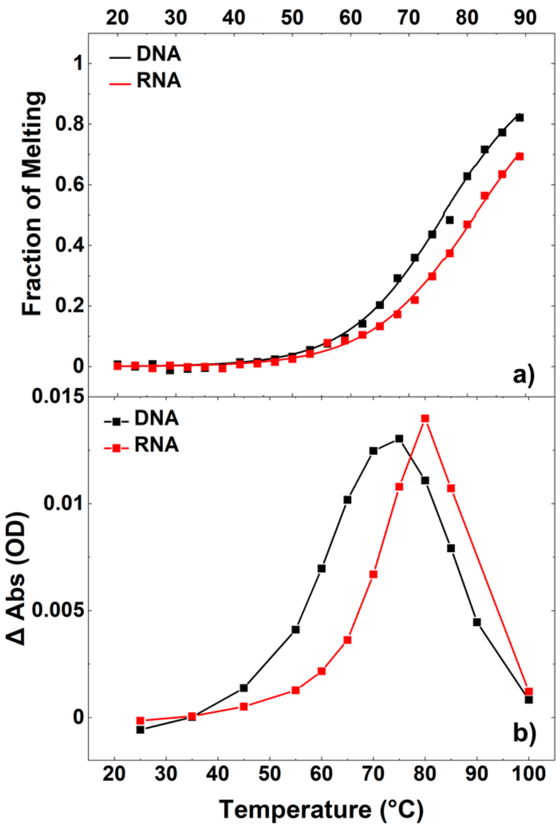

Figure S2. RNA and DNA temperature dependent behavior shown by a) FT-IR melting curves for RNA (red) and DNA (black) extracted from the G_R mode (1575 cm^{-1}) along with sigmoidal fits (lines). Both datasets are normalised to their sigmoid fit maximum to show fraction of melting. b) The temperature dependence of the T-jump signal (T_0+5 °C) at the T-jump delay corresponding to the signal maximum (20 µs for RNA, 6 µs for DNA). FT-IR and T-jump curves are in agreement with each other for both RNA and DNA showing that T-jump and FTIR data are probing the same physical process. FT-IR curves have been baseline corrected by subtracting the 1750 cm^{-1} behavior, which is in a region with no spectral features, and subtracting a linear temperature baseline extrapolated from the 20 – 40 °C range where no melting occurs.

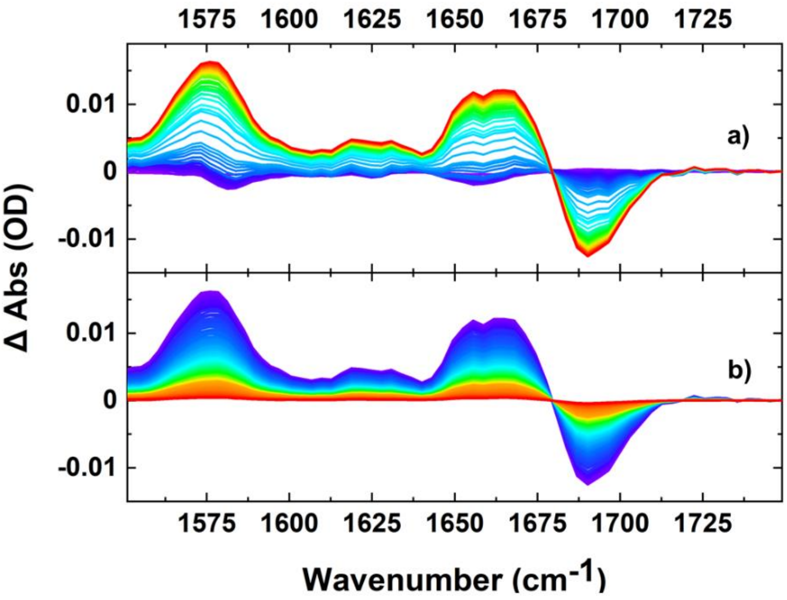

Figure S3. T-jump spectra for the RNA hairpin at a T_0 of 75 °C showing changes due to a) melting, showing data at T-jump-probe delay times between 1 ns to 20 µs (blue to red) and b) refolding, showing data obtained at T-jump-probe delay times between 20 µs and 4 ms (blue to red). It is clear that the refolding process reverses the spectral changes that occur during melting. For visual clarity, T-jump spectra have been baseline corrected by subtracting the 1750 cm^{-1} time-dependent response.

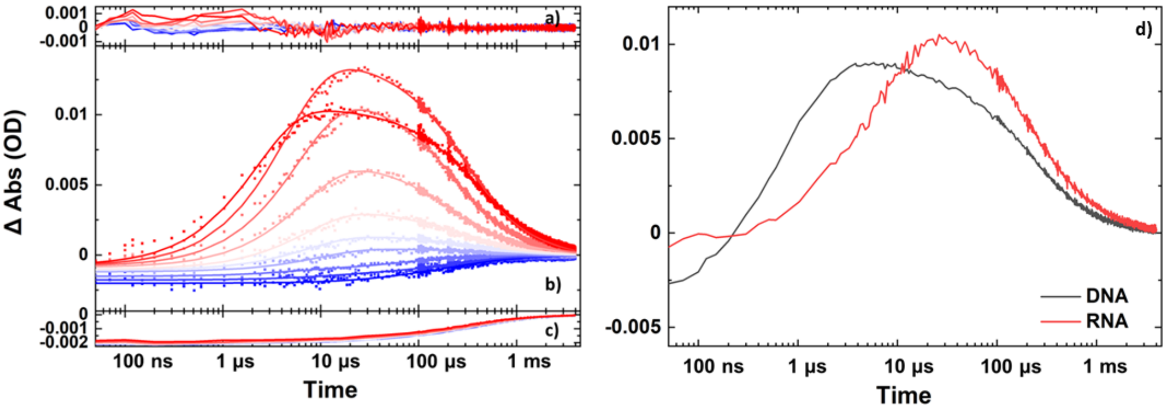

Figure S4. T-jump dynamics showing a&b) Temperature and time-dependence of the G_R band of the RNA hairpin (dots). Data are shown from a T_0 of 20 °C to 80 °C (blue-red) along with the triple exponential fits (lines). Temperature dependent fit residuals are shown in a). c) Temperature and time-dependence of the solvent dynamics from a T_0 of 20 °C to 80 °C (blue-red). All data are shown on the same scale. The low temperature RNA hairpin T-jump data, well away from the melting transition, shows only solvent dynamics. No solvent correction was applied as the solvent contribution is small (~15% of the signal) and thus does not contribute materially to the temperature dependent behavior. d) Comparison of DNA and RNA Kinetics for a T_0 of 70 °C. This is T_m – 5 for DNA and T_m – 10 for RNA.

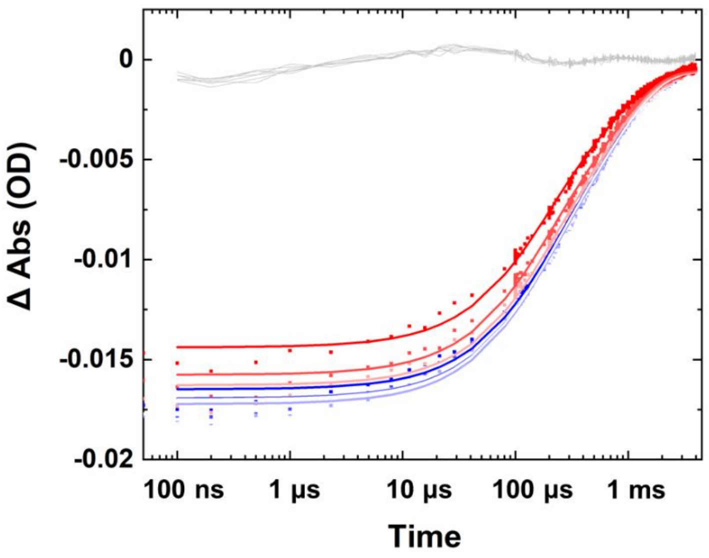

Figure S5. Temperature dependent T-jump spectroscopy data showing the temporal response of the carboxyl stretching vibrational mode of trifluoroacetic acid (TFA, dots) along with fits to bi-exponential functions (lines), at T_0 values from 20 to 80 °C (blue to red). Fit residuals are shown as grey lines. The TFA peak has been shown to provide an effective "thermometer" to determine the size of the T-jump and track dynamics of the D_2O.[5] Fits were conducted on the raw data starting from 100 ns to avoid the initial T-jump rise; no baseline or solvent correction was performed.

Table S1: Triple exponential fitting parameters for the time dependence of the G_R (1575 cm^{-1}) band of RNA and DNA hairpins over a range of T_0 values (65-80 °C and 60-80 °C respectively). Fitting was performed over the time range -20 ns to 4 ms. The results for the TFA calibration sample show the outcome of fitting to a double exponential function to the time dependence of the carboxylate band at 1670 cm^{-1}. Fitting for TFA data was performed from 100 ns to 4 ms. The data in the Table represent the result of an individual measurement at each starting temperature. By contrast, the data shown in the main text (Fig 3(b)) represent the average of repeated measurements.

	T (°C)	A_1 (OD)	A_2 (OD)	A_3 (OD)	τ_1 (μs)	τ_2 (μs)	τ_3 (μs)	Y_0 (OD)
**RNA**	65	-0.0077	0.0049	0.0018	6.9	199	1080	0.0142
	70	-0.0123	0.0083	0.0030	6.1	220	1070	0.0236
	75	-0.0149	0.0102	0.0037	4.5	283	1260	0.0287
	80	-0.0114	0.0070	0.0034	2.3	350	1530	0.0216
**DNA**	60	-0.0099	0.0050	0.0017	1.5	135	583	0.0167
	65	-0.0117	0.0062	0.0025	1.1	169	716	0.0203
	70	-0.0118	0.0062	0.0026	0.8	220	920	0.0205
	75	-0.0097	0.0051	0.0017	0.6	298	1350	0.0164
	80	-0.0067	0.0034	0.0012	0.5	449	8370	0.0120
**TFA**	70		0.0067	0.0085		149	757	0.0005

References:

(1) Banyay, M.; Sarkar, M.; Gräslund, A. A Library of IR Bands of Nucleic Acids in Solution. *Biophys. Chem.* **2003**, *104*, 477–488.

(2) Lee, C.; Park, K. H.; Cho, M. Vibrational Dynamics of DNA. I. Vibrational Basis Modes and Couplings. *J. Chem. Phys.* **2006**, *125*, 114508.

(3) Lee, C.; Cho, M. Vibrational Dynamics of DNA. II. Deuterium Exchange Effects and Simulated IR Absorption Spectra. *J. Chem. Phys.* **2006**, *125*, 114509.

(4) Greetham, G. M.; Donaldson, P. M.; Nation, C.; Sazanovich, I. V.; Clark, I. P.; Shaw, D. J.; Parker, A. W.; Towrie, M. A 100 KHz Time-Resolved Multiple-Probe Femtosecond to Second Infrared Absorption Spectrometer. *Appl. Spectrosc.* **2016**, *70*, 645–653.

(5) Fritzsch, R.; Greetham, G. M.; Clark, I. P.; Minnes, L.; Towrie, M.; Parker, A. W.; Hunt, N. T. Monitoring Base-Specific Dynamics during Melting of DNA-Ligand Complexes Using Temperature-Jump Time-Resolved Infrared Spectroscopy. *J. Phys. Chem. B* **2019**, *123*, 6188–6199.

(6) Greetham, G. M.; Clark, I. P.; Young, B.; Fritsch, R.; Minnes, L.; Hunt, N. T.; Towrie, M. Time-Resolved Temperature-Jump Infrared Spectroscopy at a High Repetition Rate. *Appl. Spectrosc.* **2020**, *74*, 720–727.

(7) Minnes, L.; Greetham, G. M.; Shaw, D. J.; Clark, I. P.; Fritzsch, R.; Towrie, M.; Parker, A. W.; Henry, A. J.; Taylor, R. J.; Hunt, N. T. Uncovering the Early Stages of Domain Melting in Calmodulin with Ultrafast Temperature-Jump Infrared Spectroscopy. *J. Phys. Chem. B* **2019**, *123*, 8733–8739.
